# Spatio-Temporal Bone Remodeling after Hematopoietic Stem Cell Transplantation

**DOI:** 10.3390/ijms22010267

**Published:** 2020-12-29

**Authors:** Constanze S. Schwarz, Christian H. Bucher, Claudia Schlundt, Sarah Mertlitz, Katarina Riesner, Martina Kalupa, Lydia Verlaat, Oskar Schmidt-Bleek, Radost A. Sass, Katharina Schmidt-Bleek, Georg N. Duda, Olaf Penack, Il-Kang Na

**Affiliations:** 1 Department of Hematology, Oncology and Tumor Immunology, Charité—Universitätsmedizin Berlin, 10117 Berlin, Germany; constanze.schwarz2@charite.de (C.S.S.); sarah.mertlitz@charite.de (S.M.); katarina.riesner@charite.de (K.R.); martina.kalupa@charite.de (M.K.); lydia.verlaat@charite.de (L.V.); olaf.penack@charite.de (O.P.); il-kang.na@charite.de (I.-K.N.); 2 Berlin Institute of Health, 10178 Berlin, Germany; 3 Berlin Institute of Health Center for Regenerative Therapies, Charité—Universitätsmedizin Berlin, 10117 Berlin, Germany; christian.bucher@charite.de (C.H.B.); claudia.schlundt@charite.de (C.S.); oskar.schmidt-bleek@charite.de (O.S.-B.); radost.sass@charite.de (R.A.S.); georg.duda@charite.de (G.N.D.); 4 Julius Wolff Institute and Center for Musculoskeletal Surgery, Charité—Universitätsmedizin Berlin, 10117 Berlin, Germany; 5 Experimental and Clinical Research Center, 13125 Berlin, Germany

**Keywords:** hematopoietic stem cell transplantation, T lymphocytes, bone remodeling

## Abstract

The interaction of hematopoietic cells and the bone microenvironment to maintain bone homeostasis is increasingly appreciated. We hypothesized that the transfer of allogeneic T lymphocytes has extensive effects on bone biology and investigated trabecular and cortical bone structures, the osteoblast reconstitution, and the bone vasculature in experimental hematopoietic stem cell transplantations (HSCT). Allogeneic or syngeneic hematopoietic stem cells (HSC) and allogeneic T lymphocytes were isolated and transferred in a murine model. After 20, 40, and 60 days, bone structures were visualized using microCT and histology. Immune cells were monitored using flow cytometry and bone vessels, bone cells and immune cells were fluorescently stained and visualized. Remodeling of the bone substance, the bone vasculature and bone cell subsets were found to occur as early as day +20 after allogeneic HSCT (including allogeneic T lymphocytes) but not after syngeneic HSCT. We discovered that allogeneic HSCT (including allogeneic T lymphocytes) results in a transient increase of trabecular bone number and bone vessel density. This was paralleled by a cortical thinning as well as disruptive osteoblast lining and loss of B lymphocytes. In summary, our data demonstrate that the adoptive transfer of allogeneic HSCs and allogeneic T lymphocytes can induce profound structural and spatial changes of bone tissue homeostasis as well as bone marrow cell composition, underlining the importance of the adaptive immune system for maintaining a balanced bone biology.

## 1. Introduction

Primary immunodeficiency diseases, the rescue of bone marrow (BM) aplasia following high-dose chemotherapy for lymphoma and multiple myeloma or stem cell-derived hematological malignancies such as acute leukemia require hematopoietic stem cell transplantation (HSCT), usually derived from BM, peripheral blood, or umbilical cord blood. The transplanted cells can either be of autologous (patient derived), allogeneic (donor derived) or syngeneic (identical twin derived) origin. However, delayed immune reconstitution or hampered immune competence after HSCT can lead to high risk infections and tumor relapse [1,2]. In the setting of allogeneic HSCT (allo-HSCT) acute graft-versus-host disease (aGVHD) is an additional major, survival-limiting complication, mediated by donor T lymphocytes attacking the recipient’s tissue. Allo-HSCT requires allele level HLA (human leukocyte antigen) matching between the donor and recipient [3,4]. HLA typing serves to determine the histocompatibility of donor cells, which is critical for the success of allo-HSCT. HLA molecules are encoded by many genes in the MHC region (major histocompatibility complex). HLA molecules of the donor and recipient are matched as closely as possible to reduce the risk of a rejection reaction (host-versus-graft) and graft-versus-host. HLA typing may reduce the risk but since only 25–30% of patients can find an HLA matched familial donor (HLA identical siblings), most allogeneic transplants are performed using grafts from unrelated donors [4]. Acute GVHD is an inflammatory disease that is unique to allo-HSCT due to the recognition of the recipient’s tissues by transplanted donor immune cells. Target organs of aGVHD such as skin, gastrointestinal tract and liver can be affected to varying degrees [5,6,7]. Interestingly, previous studies showed a higher risk of fractures after HSCT compared to healthy untreated individuals, whereby the causes are probably multifactorial [8,9,10].

Hematopoiesis, including immune cell development, such as B lymphopoiesis, occurs in the BM and it has been demonstrated that bone elements are actively participating in the regulation of hematopoiesis and immunity [11,12]. Only recently, we and others have been able to demonstrate that a tight interplay between hematopoietic stem cells and immune cells residing in the BM is essential to maintain bone homeostasis and tissue structure [13,14]. However, details and consequences of this interplay are not yet completely understood. To shed more light on the effect of immune cells on bone structural organization, we decided to use a murine model of allo-HSCT. Following allo-HSCT, donor T lymphocytes infiltrate the BM microenvironment making it an ideal setting to further study the interplay of adaptive immune cells and bone as an organ. Recent studies demonstrated niche-forming cells in the BM as targets of GVHD and described an increased incidence of osteopenia, osteoporosis and bone fractures after allo-HSCT [8,15,16]. We recently found that BM infiltrating allogeneic donor T lymphocytes are associated with loss of osteoblasts in allo-HSCT patients and hampered B cell regeneration in GVHD patients [17,18].

The aim of this study was to unravel the influence of allogeneic T lymphocytes in an allo-HSCT setting during aGVHD on the trabecular and cortical bone structures, the osteoblast reconstitution, and the bone vasculature.

## 2. Results

### 2.1. Bone Marrow Infiltration of Donor T Lymphocytes after Allo-HSCT

**Figure 1:**
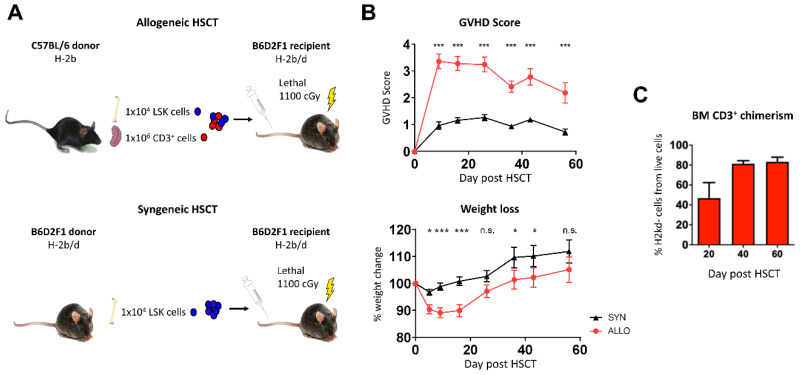
(**A**) Overview of the experimental hematopoietic stem cell transplantation protocol: allogeneic setting and syngeneic setting. (**B**) Clinical data of syn- and allo-HSCT mice (syn N = 19, allo N = 21). (**C**) Chimerism analysis of BM CD3^+^ cells in allo-HSCT mice (N = 6–7 per date). Error bars indicate mean ± SEM; * *p* ≤ 0.05; *** *p* ≤ 0.001; n.s. not significant by unpaired Student’s T-test.

To investigate the influence of aGVHD on the cortical and trabecular bone structure, osteoblast reconstitution and bone vasculature, we used a clinically relevant haplo-identical murine allo-HSCT model (Figure 1A). The graft consisted of a defined number of Lineage^−^Sca-1^+^c-Kit^+^ (LSK) BM cells (1 × 10^4^) and mature splenic cluster of differentiation (CD) 3^+^ T lymphocytes (1 × 10^6^). The donor LSK cells are essential for immune reconstitution of the host after lethal irradiation as conditioning therapy, since the hematopoietic cells of the host are fully depleted by the lethal irradiation. The donor T lymphocytes mediate the aGVHD.

The set-up obtained a typical clinical phenotype with increasing aGVHD scores and decreasing weight in allo-HSCT recipients (Figure 1B).

To verify BM involvement in aGVHD in our C57BL/6 → B6D2F1 mouse model we studied donor T cells in the BM post-transplant. T cells with donor chimerism were only detectable in the allo-HSCT mice and the percentage of donor T cells was already 46% ± SEM at day +20 after allo-HSCT (Figure 1C).

An important feature of the herein described aGVHD model is the LSK cell selection of the donor BM cells, thereby allowing the transplantation of purified hematopoietic stem and progenitor cells along with a defined number of donor mature T cells which mediate aGVHD. This selection enabled us to specifically analyze and correlate the remodeling of the host bone by allogeneic T cells during aGVHD.

### 2.2. Increased Trabecular Bone Number and Decreased Cortical Thickness in aGVHD

**Figure 2:**
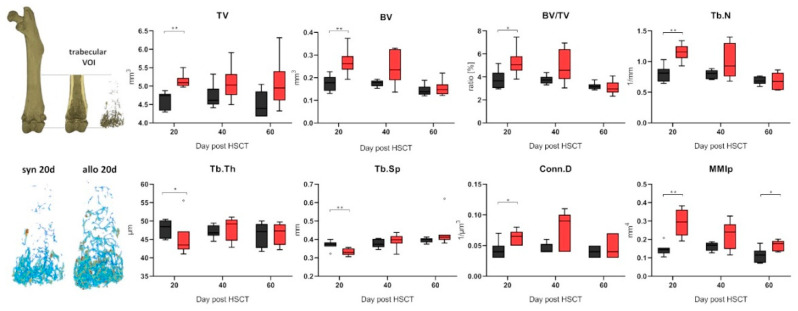
Analyzing the trabecular bone in the distal femora shaft (trabecular VOI is depicted above left) revealed significant differences within the total volume (TV), the bone volume (BV) and the ratio thereof (BV/TV) (syn N = 7, allo N = 6). The trabecular separation (Tb.Sp), number (Tb.N) and connectivity between individual structures (Conn.D) was also significantly altered and the trabecular thickness (Tb.Th) was distinctly lower after allo-HSCT. The polar moment of inertia (MMIp) was significantly increased after allo-HSCT. Shown are representative images. * *p* ≤ 0.05; ** *p* ≤ 0.01 by Mann-Whitney-U-Test.

To identify changes in the bone substance we performed microCT analyses of the bones after HSCT. We found distinct differences in the trabecular bone structure between allo-HSCT and syngeneic HSCT (syn-HSCT) recipients (Figure 2). Allo-HSCT led to higher bone volume in the trabecular area and to higher trabecular numbers compared to the syn-HSCT group at day +20 post transplantation. Significantly higher trabecular numbers were accompanied by significantly lower trabecular separation and increased connectivity between trabecular structures while the trabecular thickness had decreased, indicating a de novo formation of trabecular branches. The volumetric isolated polar moment of inertia significantly increased due to higher branching of trabecular structures (Figure 2). Strikingly, these changes occurred in a relatively short time period till day +20 after allo-HSCT (Figure 2).

**Figure 3:**
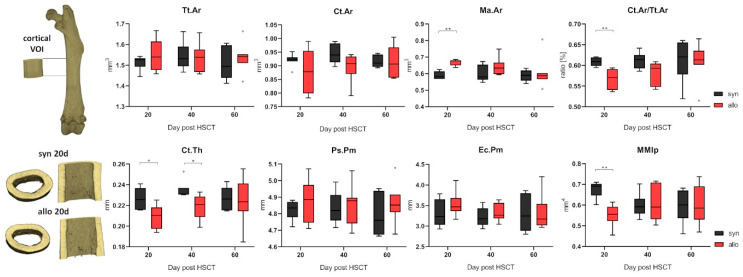
Cortical bone structure was analyzed in the midshaft of the femora (cortical VOI as indicated above left; allo N = 6, syn N = 7). While the periosteal perimeter (Ps.Pm) and the total area (Tt.Ar) of the bone remained unchanged, the cortical thickness (Ct.Th) diminished in allo-HSCT recipients. The cortical thinning was caused by a significantly larger medullary area (Ma.Ar) and the significantly lower ratio of total and cortical area (Ct.Ar/Tt.Ar), however the endocortical perimeter (Ec.Pm) was only slightly increased. The polar moment of inertia (MMIp) was decreased due to the thinner cortical bone in allo-HSCT recipients. * *p* ≤ 0.05; ** *p* ≤ 0.01 by Mann-Whitney-U-Test.

Analyzing the cortical bone in the diaphyseal midshaft of the femora of allo-HSCT recipients at day +20 post transplant revealed a significant change in the medullary cavity indicating bone remodeling (Figure 3). The larger medullary area was accompanied with a significantly thinner cortical bone in allo-HSCT as compared to syn-HSCT recipients, and this cortical thinning resulted to a significantly lower ratio of cortical to total bone area in the diaphyseal femoral bone and to a significant decrease in the polar moment of inertia. These structural changes indicate major remodeling processes in bone quality and quantity in the early phase after allo-HSCT and could be speculated as an assignable influencing factor for the increased fracture rates post-transplantation observed in patients in the early phase after allo-HSCT.

Based on the observed changes in the mineralized bone tissue, we further performed histological analyses of osteogenic cells for possible effects of allogeneic T lymphocytes on the bone forming cells.

### 2.3. Disruptive Osteoblast Lining in Bones during aGVHD

**Figure 4:**
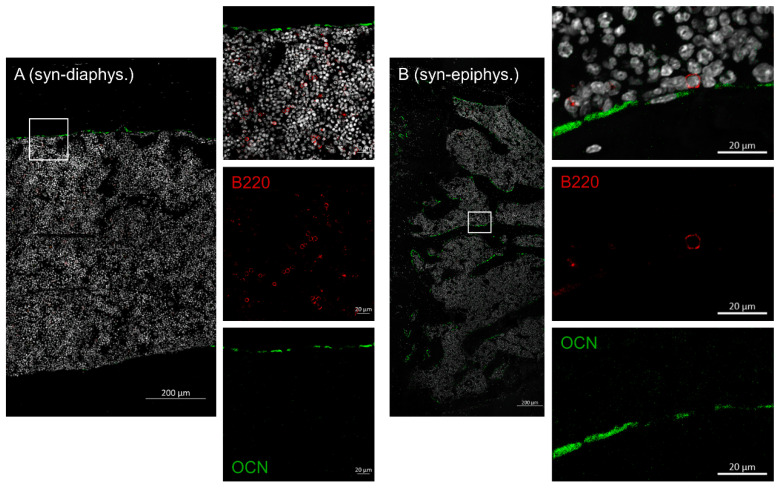
Femora harvested at day +20 after syn-HSCT were prepared for immunohistochemistry (N = 6). Within the diaphysis of the femora ((**A**) syn-diaphys.) osteoblasts line the endosteal region while B cells are evenly distributed within the marrow cavity, the same result was true for the epiphyseal bone ((**B**) syn-epiphys.). Nuclei (Dapi = white, 2nd outtake image from top to bottom), B cells (B220 = red, single stain 3rd outtake image from top to bottom), osteoblasts (osteocalcin = green, single stain 4th outtake image from top to bottom). Representative images are shown.

Next, we were interested in B cell numbers in the BM and if the osteoblasts were physiologically lining up at the interface of bone and BM after allo-HSCT. Femora from day +20 after HSCT (allo vs. syn-HSCT) were harvested and stained with osteocalcin (osteoblasts) and B220 (B cells). In bones of syn-HSCT recipients, osteoblasts were found in a physiological lining cell configuration in the endosteal regions in the diaphysis as well as the epiphyseal bone regions (Figure 4). B cells were evenly distributed throughout the BM, in part found in direct contact with the osteoblasts.

**Figure 5:**
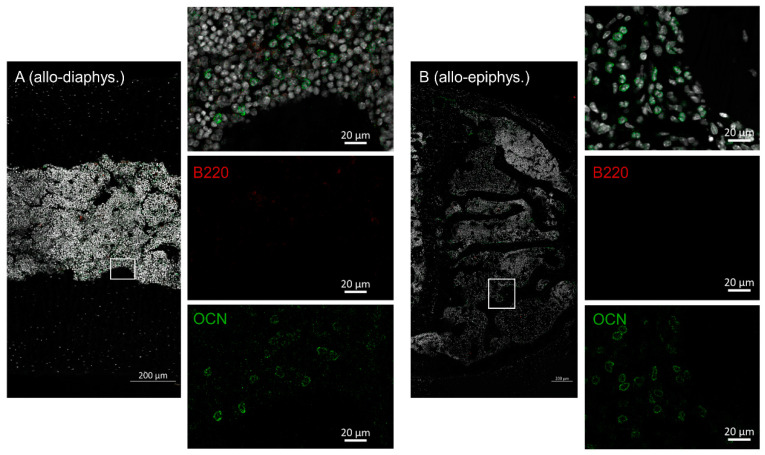
Femora harvested at day +20 after allo-HSCT were prepared for immunohistochemistry (N = 6). Within the diaphysis of the femora ((**A**) allo-diaphys.) no osteocalcin positive lining cells were detected within the endosteal region and B cells were absent from the BM cavity. Of note, osteoblasts were found evenly distributed throughout the BM space. The same result was true for the epiphyseal bone ((**B**) allo-epiphys.). Nuclei (Dapi = white, 2nd outtake image from top to bottom), B cells (B220 = red, single stain 3rd outtake image from top to bottom), osteoblasts (osteocalcin = green, single stain 4th outtake image from top to bottom). Representative images are shown.

In sharp contrast, bone samples of allo-HSCT recipients presented with significantly altered cellular distribution. Osteoblast lining was not detectable at all, although osteocalcin positive cells were distributed throughout the BM cavity (Figure 5). B cells were also completely lacking. We found these altered cellular patterns in the diaphysis as well as in the epiphyseal bone region.

### 2.4. Decreased Osteoid Lining in Diaphyseal Region of Bones in aGVHD

To investigate the association of osteoblast redistribution and bone formation, we stained osteoid in the epiphyseal and diaphyseal region of bones. Femora were harvested at day +20 after allo-HSCT and stained with von Kossa and van Gieson staining.

**Figure 6:**
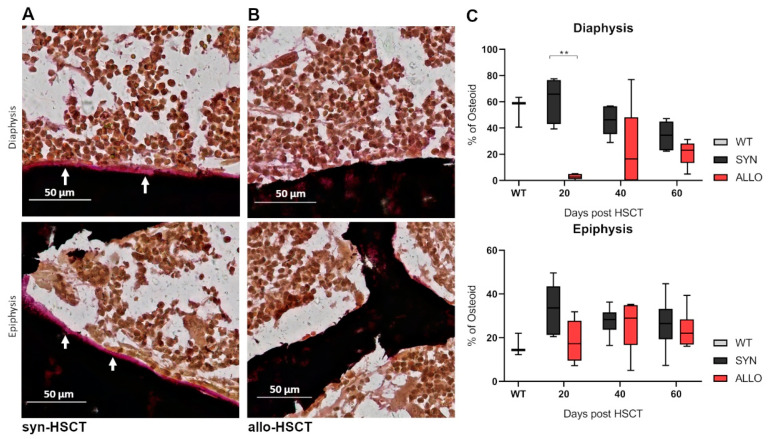
Representative images of femora of HSCT recipients after histological visualization by von Kossa and van Gieson staining (N = 3). Mineralized bone tissue (black), BM (orange-red) and osteoid (pink) are visualized. (**A**) Epiphyseal and Diaphyseal region of the femora from syn-HSCT recipients. (**B**) Epiphyseal and Diaphyseal region of the femora from allo-HSCT recipients. Scale bar = 50 µm. (**C**) Percentage of osteoid analyzed in the diaphyseal and epiphyseal region of femora (WT N = 3, syn N = 6, allo N = 6). ** *p* ≤ 0.01 by Mann-Whitney-U-Test.

Syn-HSCT recipients showed a lining of osteoid in the endosteal region of the diaphyseal and epiphyseal region of the bone (Figure 6A). In contrast, osteoid lining in the endosteal region of the diaphyseal bone was significantly reduced in allo-HSCT recipients (Figure 6B,C).

### 2.5. Increased BM Vessel Density in aGVHD

**Figure 7:**
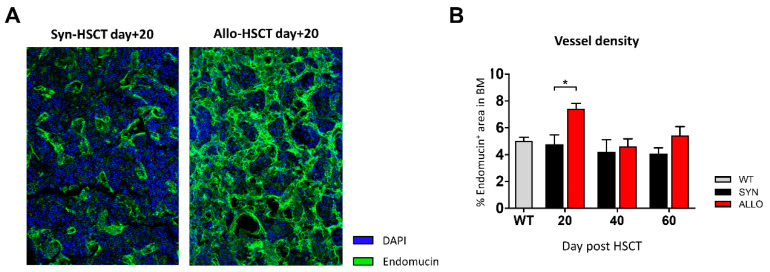
(**A**) Representative pictures of BM sections stained with Endomucin (green) on day +20 after HSCT. Nuclei were counterstained with Dapi (blue). (**B**) Analysis of vessel density in the BM of BDF syn- and allo-HSCT mice (WT N = 3, syn N = 7, allo N = 6). Error bars indicate mean ± SEM; * *p* ≤ 0.05 by unpaired Student’s T-test.

Since the vasculature is critical for bone development and has been demonstrated to be involved in aGVHD pathobiology, we were interested in the vessel density in the BM during aGVHD. To study vasculature kinetics post allo-HSCT, we performed staining with the endothelial marker endomucin (Figure 7A). At day +20 post transplantation, we found that allo-HSCT recipients with aGVHD had significantly higher vessel density in femoral bones as compared with syn-HSCT recipients without aGVHD (Figure 7B). However, in the follow-up 40 and 60 days post HSCT, no differences between both groups could be observed (Figure 7B). The pictures in panel A demonstrate typical examples of normal BM vasculature after syn-HSCT (left) and increased BM vascular density after allo-HSCT (right). Quantification is given in panel B of Figure 7.

## 3. Discussion

The bone is of major interest after allo-HSCT because of its critical importance for hematopoiesis, and immunity but also niche for hematological malignancies. It is well known that bone substance loss is a late complication, years after allo-HSCT, due to multiple factors, including hormonal changes [10,19]. Surprisingly, little is known regarding the influence of allo-HSCT on the bone structure at earlier time points after allo-HSCT. We found that infiltration of donor T lymphocytes was associated with significant alteration of the bone structure, BM vasculature and osteoblast reconstitution in the early phase after allo-HSCT. To our knowledge this is the first publication on investigation of the bone structure early after allo-HSCT.

We found that trabecular bone substance was increased during aGVHD after allo-HSCT, whereas cortical thickness was reduced. Changes in the microstructure of the bone, like a decreased cortical thickness and increased trabecular number and branching, alters the mechanical stability of the femora [20]. A cortical bone loss could be observed before in patients with non-radiographic axial spondyloarthritis and chronic kidney diseases as well as in a murine model of systemic lupus erythematosus [21,22,23]. However, in those models, the bone remodeling and loss in biomechanical competence was uncompensated, and therefore the trabecular bone structures remained unaffected, except in patients with chronic kidney diseases where a slight increase in trabecular number could be observed [21,22,23]. It can be assumed that the changes in the BM and the increase in trabecular bone with simultaneous loss of cortical thickness after allo-HSCT are associated with increased bone turnover. Surprisingly, the load bearing capacity of the bone shifted from the cortical wall towards trabecular structures since an increase in trabecular number and connectivity between the trabeculae could be found, whereas the cortical wall was found to be thinner. Interestingly, processes of bone loss, which are associated with advancing age, glucocorticoid excess or atherosclerosis, seem to follow a different mechanism with decreased trabecular bone and increased cortical bone [24,25,26]. In aged individuals, the increased cortical thickness leads to lesser trabecular bone, however this shift in bone thickness leads to a more brittle bone [27]. A thinner cortical bone with higher trabecular structures leads to a reduced fracture competence compared to a brittle fracture manner in bones with a high cortical thickness. This is an example of the principle that the form is adapting to the function. We showed an increase in the polar moment of inertia, an indicator of the resistance to torsional stress, in the trabecular structure and a decrease in the cortical structure, a process distinctly different to an age associated alteration of bone.

In physiological conditions, osteoblasts and osteoclasts maintain the bone homeostasis and further the mass and structural integrity of the skeleton by constantly remodeling the bone according to the mechanical cues of loading. Changes in the bone structure imply changes in the maintenance of the mineralized matrix. The distribution of osteocalcin positive osteoblastic cells in the diaphyseal and endosteal areas of the femora were significantly altered in allo-HSCT recipients with disrupted bone lining of osteoblasts, and displacement of osteocalcin positive cells to the BM. This indicated that no bone deposition occurred on the endosteal bone surface, which would correspond with the decreasing cortical bone thickness. Furthermore, the redistribution of osteoblasts led to a decrease of osteoid formation. The first step in forming new bone is osteoid deposition. A decrease in the production of osteoid leads to the suggestion that a decrease in bone formation takes place. It is known that the osteoblast lineage supports the maintenance of B lymphopoiesis [28]. Upon analyzing the distribution of B cells in the BM, it became apparent that syn-HSCT mice showed a normal distribution also seen in mice without HSCT, while allo-HSCT mice lacked B cells among the BM cells at day +20 after transplantation. Several mouse models have shown that BM B lymphopoiesis is impaired by allogeneic T lymphocytes [16,29,30,31]. Thus, the changes in the osteoblastic cell distribution could be causative for the lacking B220 positive B cells in the BM of allo-HSCT mice. Mensen et al. could recently translate these findings to the human setting and demonstrated a dramatic reduction of osteoblasts and delayed B-cell regeneration in allo-HSCT patients, which was significantly associated with systemic aGVHD and full-intensity conditioning therapy [17]. B cells furthermore regulate the OPG-RANKL-RANK pathway that regulates osteoclastogenesis. We found an impaired immune cell reconstitution in allo-HSCT recipients and a defective osteoclast regulation has to be expected. The changes in B cells could thus be another reason for the modifications in the bone structure during the early phases after transplantation. Our current results are in accordance with the mentioned previous publications and further highlight the role of osteoblasts after allo-HSCT. This is of particular interest in the context of previous studies providing proof of principle that osteoblasts can serve as therapeutic targets: Terashima et al. found that, during sepsis, osteoblast stimulation by parathyroid hormone (PTH) resulted in increased T and B cells in BM and peripheral blood [32]. Similarly, Matic et al. described PTH administration as a stimulator for bone lining cell activation into active osteoblasts [33].

In preclinical models as well as in patients, earlier studies could demonstrate that aGVHD is associated with the formation of new blood vessels in aGVHD target organs and that angiogenesis is primary involved in the initiation of tissue inflammation [34,35,36,37]. Here, we showed an increased BM vessel density at day +20 after allo-HSCT, underlining that the BM is an aGVHD target organ. Bone is a richly vascularized tissue and vascularization is necessary for bone development, regeneration and remodeling. It is described that angiogenesis is closely associated with bone resorption and angiogenic factors like vascular endothelial growth factor (VEGF) and endothelin, regulating osteoclast and osteoblast activity [38,39]. In addition, blood vessels transport circulating osteoblast precursors to sites undergoing active remodeling [40]. Earlier studies confirm a strong relationship between angiogenesis and osteogenesis and propose that vessel density is linked to architectural renovation in the trabecular and the cortical bone [41]. For trabecular bone formation evidence suggests the contribution of osterix expressing cells moving along with the vessels to form new bone [33]. In our findings, this is pictured by increased vessel density and increased trabecular numbers during aGVHD. Further longitudinal follow-up would be interesting for future studies. In addition, a limitation of the current study is that we included T cells in the allogeneic but not the syngeneic setting. We made this decision to reduce animal numbers and costs because we did not expect major effects of adoptively transferred syngeneic T cells on the bone structure. In future experiments the inclusion of syngeneic T cells could help discriminate more precisely between T cell effects per se and effects due to HLA incompatibility between donor and host.

For cortical bone remodeling, blood vessels follow the cutting cones and deliver nutrients and growth factors for osteoblasts to create new bone behind the cone. Concordantly, we found single osteoblasts in the BM of allo-HSCT mice, which were not seen in BM compartment of syn-HSCT mice. We hypothesize that angiogenesis is an additional component for the complex immunoskeletal interaction. Our observations could foster further experimental studies including more detailed investigations of underlying molecular mechanisms.

In conclusion, we provide novel insights into the pathophysiology of aGVHD harming the bone structure and stressing the relevance of osteoblasts as key players at the osteo–immune interface and as a therapeutic target following HSCT.

## 4. Materials and Methods

### 4.1. Mice

Animal experiments were approved by the respective regional agency (Landesamt für Gesundheit und Soziales Berlin, G0119/15 (approval letter 16 June 2015) and G0081/19 (approval letter 12 August 2019)). Female C57BL/6 (B6) (H-2b) and B6D2F1 (BDF) (H-2b/d) mice were purchased from Charles River Laboratories (Sulzfeld, Germany) and were housed in the Charité University Hospital animal facility under specific pathogen-free controlled conditions and a 12-h light/dark cycle with water and food ad libitum. Animals used in the experiments were 10 to 12 weeks old. Health monitoring was performed daily.

### 4.2. Experimental Hematopoietic Stem Cell Transplantation Protocol

Murine HSCT models are well described and have been used by many groups worldwide, including our group [34,42,43,44]. In short, the experimental HSCT protocol consists of the following steps:

(1) Donor cell isolation: On day +0 of the HSCT, donor mice (B6 for allo- and BDF for syn-HSCT) were sacrificed by cervical dislocation; bones (femora and tibiae) and spleens were harvested; LSK cells were isolated out of the BM; CD3^+^ cells were isolated out of the spleen.
(2) Conditioning: On day +0 of the HSCT, recipient mice (BDF for both groups) received myeloablative total body irradiation (TBI).
(3) Transplantation: On day +0 of the HSCT, irradiated recipient mice (BDF) were injected intravenously (i.v.) with isolated LSK cells and the allo-HSCT group additionally with CD3^+^ cells.

Figure 1A demonstrates the protocol in a simplified manner.

### 4.3. LSK Cell Isolation

BM cells from donor B6 mice (allo-HSCT) or BDF mice (syn-HSCT) were flushed out of the tibia and femur. The solution was carefully passed through a 23 G needle and over a 70 µm cell strainer (BD Biosciences, San Jose, CA, USA) and a single-cell suspension was prepared in phosphate-buffered saline (PBS)/2% fetal calf serum/1mM EDTA. Lineage depletion was obtained by Magnetic Activated Cell Sorting (MACS) separation (Miltenyi Biotec, Bergisch Gladbach, Germany), using CD5, CD45R (B220), CD11b, GR-1 (Ly-6G/C), 7-4, Ter-119 as lineage markers, and a following fluorescently activated cell sort (FACS) by marker expression Sca-1^+^ and c-Kit^+^.

### 4.4. T Cell Isolation

Spleens from B6 donor mice were grounded and passed through a 40 µm cell strainer (BD Biosciences, San Jose, CA, USA) and resuspended in MACS buffer. Splenic T cell suspension was obtained using the Pan T cell Isolation Kit II (Miltenyi Biotec, Bergisch Gladbach, Germany). The kit is based on a cocktail of biotin-conjugated antibodies against CD11b, CD11c, CD19, CD45R (B220), CD49b (DX5), CD105, Anti-MHC-class II, and Ter-119. The isolation of T cells is achieved by depletion of magnetically labeled cells. T cell purity was evaluated by CD3^+^ staining and flow cytometry analysis and ranged from 72.9% to 74%. The number of injected T cells was adjusted according to acquired purity, ranging from 1.07 × 10^6^ to 1.06 × 10^6^ cells/100 µL injection volume.

### 4.5. Conditioning and Cell Transplantation

BDF recipient mice received myeloablative total body irradiation with 1100cGy from a 137Cs source (GSR D1, Gamma Service Medical, Leipzig, Germany) as a split dose. The allo-HSCT group was subsequently injected intravenously with 1 × 10^4^ LSK cells and 1 × 10^6^ T cells with a total injection volume of 200 µL, divided into 100 µL of LSK cells and 100 µL of T cells. The syn-HSCT group was subsequently injected intravenously with 1 × 10^4^ LSK cells and PBS with a total injection volume of 200 µL, divided into 100 µL of LSK cells and 100 µL of PBS. Wild-type (WT) BDF mice did not receive irradiation or cells.

### 4.6. GVHD Monitoring

The clinical aGVHD score was assessed by the summation of individual score numbers of five clinical parameters (weight loss, posture, activity, fur texture and skin integrity) according to the established Cooke grading system [45,46]. Each clinical parameter was rated on a scale of 0–2 and mice were sacrificed when their total score exceeded 6, or if one parameter reached a score of 2. Mice were individually scored twice weekly.

### 4.7. Chimerism Analysis

Chimerism analysis was performed by H-2kb and H-2kd chimerism markers. Allogeneic donors (C57BL/6) exhibit only H-2kb, whereas syngeneic donors and recipients (B6D2F1) exhibit H-2kb/d. Allo-HSCT recipients develop a mixed chimerism indicated by donor-specific absence of H-2kd. Representative flow cytometry plots of the three timepoints are given in the supplementary material section (see Supplementary Figure S1).

### 4.8. Flow Cytometry

Cells were washed twice and stained for 20 min at 4 °C in PBS/0.5 mM EDTA/0.5% bovine serum albumin (BSA) with anti-Sca-1 (D7-FITC) rat monoclonal antibody, anti-c-Kit (2B8-APC) rat monoclonal antibody, anti-CD3 (145-2c11-PE) hamster monoclonal antibody or anti-H2kb (AF6-88.5-FITC) rat monoclonal antibody (all BD Biosciences, San Jose, CA, USA). Samples were analyzed by BD FACSCanto II (BD Biosciences, San Jose, CA, USA) and FlowJo Software (TreeStar Inc., Ashland, OR, USA).

### 4.9. X-ray Microtomographic Analysis

Harvested bones were fixated in 4% paraformaldehyde (PFA) (SAV Liquid Production, Flintsbach am Inn, Germany) solution for 4h at room temperature and thoroughly rinsed with water before undergoing X-ray microtomography (microCT) analysis in a custom-made fixation device. Bones were scanned with a nominal resolution of 10 µm in a SkyScan 1172 high-resolution microCT (Bruker, Kontich, Belgium). A 0.5 mm aluminum filter and an X-ray tube voltage of 80 kV, 124 µA, and maximized power were employed. Image acquisition was done with a 180° orbital scan in 0.2° rotation step and camera pixel binning for higher sensitivity. The shadow images were reconstructed with a modified Feldkamp algorithm (NRecon software, Bruker, Kontich, Belgium) applying Gaussian smoothing, ring artefact reduction, misalignment compensation, and beam hardening correction.

The diaphyseal bone was analyzed applying a global threshold selected via a mineral density value of 590 mg/cm^3^ calcium hydroxyapatite, calibrated with reference phantoms containing defined concentrations of calcium hydroxyapatite with a similar diameter to the scanned mice bones. For the trabecular bone an adaptive thresholding was applied to minimize partial volume effects and thickness biasing. The trabecular bone was analyzed in a volume of interest (VOI) of 5.2 mm beginning 0.4 mm above the distal growth plate. The cortical VOI was analyzed 4 mm cranial from the distal growth plate with a height of 1.6 mm.

### 4.10. Histology of Murine Bones—Osteoblast and B Cell Staining

In order to analyze the distribution of B cells and osteoblasts, harvested bones were cryo-embedded according to the Kawamoto method [47]. Bones were fixated in 4% PFA solution for 4h at room temperature before they went through increasing sucrose solutions (10%, 20%, 30%) for 24 h each at 4 °C. Consequently, bones were embedded in SCEM (SectionLab, Hiroshima, Japan) in pre-cooled n-hexane and were kept at −80 °C. Then, thin serial sections of 7 µm were created with a cryostat (Leica, Wetzlar, Germany). Immune-histological analysed frozen sections were thawed and rehydrated in PBS for 20 min. Followed by blocking for 1h with wash buffer (1X TBS + 5% FCS + 0.1% Tween-20) plus 10% donkey serum and subsequently stained with anti-osteocalcin (Enzo Life Sciences, Lörrach, Germany, final dilution 1:4000 for 1 h at room temperature). After washing, the donkey anti-rabbit AF647 antibody (BioLegend, San Diego, CA, USA) was applied for 1 h, followed by washing and blocking with wash buffer containing 10% rat serum for 1 h. Next, the anti-B220 antibody (in house production of the Deutsches Rheuma-Forschungszentrum, Berlin, Germany) was applied for B cell detection. Using DAPI to stain the cell nuclei (1:1000) for 7 min at room temperature the staining was finalized with a washing step in PBS before embedding. As control for the B cell staining, femora were stained without the inclusion of the anti-B220 antibody and as control for the osteoblast staining, the anti-osteocalcin antibody was omitted, and femora were only stained for the secondary antibody (see Supplementary Figure S2).

### 4.11. Histology of Murine Bones—Osteoid Staining

To detect the presence of osteoid built by osteoblasts, von Kossa staining coupled with van Gieson staining was done. Sections of cryo-embedded bones underwent a freeze-drying step for 24 h at −20 °C followed by storage at −80 °C. Frozen sections were fixated for 30min in 4% PFA. After washing in deionized water (dH_2_O) for 5 min, incubation with 3% silver nitrate (Merck, Darmstadt, Germany) for 10 min stained mineralized tissue followed by washing with dH_2_O. Furthermore, incubation of 2 min in 5% sodium carbonate (Merck, Darmstadt, Germany) with 10% formaldehyde followed by 10 min washing with tap water was performed. Afterwards sections were incubated in 5% sodium thiosulfate (Merck, Darmstadt, Germany) for 5 min with subsequent washing in tap water for 10 min and shortly in dH_2_O. Nuclei were stained for 15 min with iron hematoxylin after Weigert (Waldeck, Münster, Germany) followed by washing for 10 min with tap water. Consequently, sections were stained with picrofuchsin after van Gieson (Waldeck, Münster, Germany) for 3 min. To negotiate the staining sections incubated in increasing ethanol concentrations before embedding.

### 4.12. Histology of Murine Bones—Endomucin Staining

Tissue samples were fixed in 4% PFA overnight, decalcified in in 0.5 M EDTA in H_2_O at 4 °C, pH 8 for 3 days, stored in 20% Sucrose/2% polyvinylpyrrolidone (PVP)/H_2_O overnight and embedded in a solution of 8% gelatin/20% sucrose/2% PVP in H_2_O. Sections measuring 60 μm were permeabilized in 0.5% Triton-X100 in H_2_0, blocked in 5% donkey serum/0.5% Triton X-100 in H_2_0 and stained over night at 4 °C with primary goat monoclonal antibody against endomucin (PA5-47648, 1:200, Thermo Fisher Scientific, Waltham, MA, USA). Sections were stained with secondary donkey anti-goat antibody conjugated with Cy3 (Sigma-Aldrich, St. Louis, MO, USA). For Z-stacks, eight representative pictures of different BM areas were analyzed with a Zeiss ApoTome.2 microscope (Carl Zeiss Microscopy, Thornwood, NY, USA) and 35 images per Z-stack were taken. Vessel density was measured by determining endomucin positive area of each high-power field. Area was assessed by quantification of endomucin positive area to total area with a predetermined threshold using Fiji Software. As control for the endomucin vessel staining, femora were stained without the inclusion of the primary anti-endomucin antibody, and femora were only stained for the secondary antibody (see Supplementary Figure S3)

### 4.13. Statistics

For statistical analysis, unpaired Student’s T-test was used. Values are presented as mean ± SEM, values of *p* ≤ 0.05 were considered statistically significant. For the statistical analyses of the microCT data, the Mann–Whitney U test for unpaired samples lacking normal distribution was applied and a value of *p* < 0.05 (exact, 2-tailed) was considered significant. All statistical analyses were performed using GraphPad Prism software (GraphPad Software, La Jolla, CA, USA).

## Data Availability

The data presented in this study are available on request from the corresponding author.

## Supplementary Materials

Supplementary materials can be found at https://www.mdpi.com/1422-0067/22/1/267/s1.
