# Supplementary material for: Spatio-Temporal Bone Remodeling after Hematopoietic Stem Cell Transplantation
Source: Int J Mol Sci. 2020 Dec 29;22(1):267. doi: 10.3390/ijms22010267 (PMC7795370; doi:10.3390/ijms22010267)
Supplement: Supplementary file 1 [file ijms-22-00267-s001.pdf]

Supplementary Material

Spatio-Temporal Bone Remodeling after Hematopoietic Stem Cell Transplantation

Constanze S. Schwarz, Christian H. Bucher, Claudia Schlundt, Sarah Mertlitz, Katarina Riesner, Martina Kalupa, Lydia Verlaat, Oskar Schmidt-Bleek, Radost A. Sass, Katharina Schmidt-Bleek, Georg N. Duda, Olaf Penack and Il-Kang Na

1 Supplementary Figures

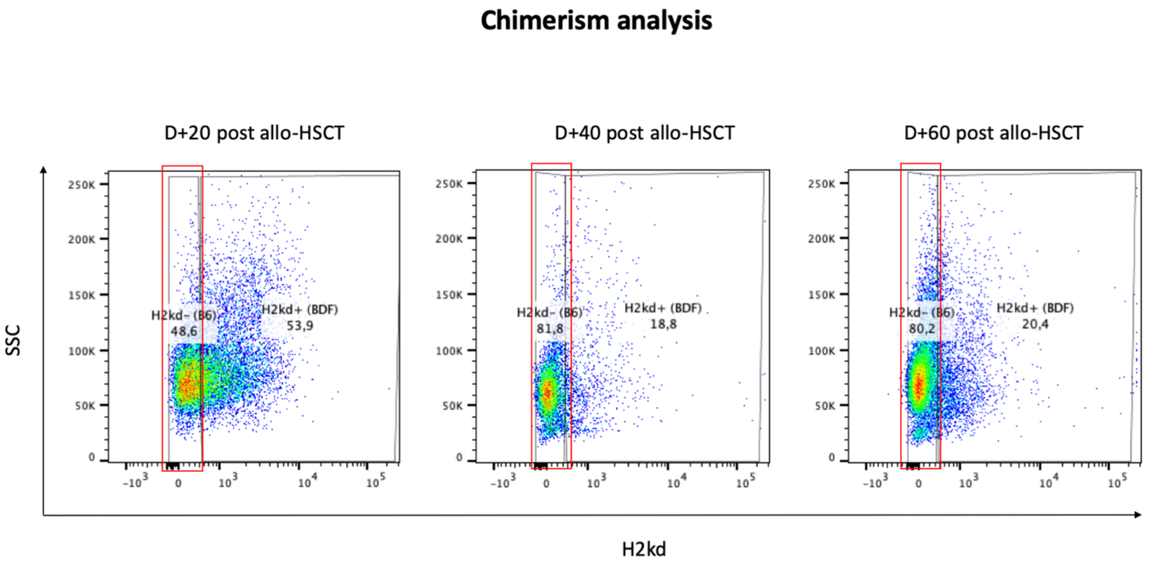

Supplementary Figure S1. Chimerism analysis of allogeneic transplanted mice over time. Chimerism is indicated by donor-specific absence of H-2kd, as allogeneic donors (B6) exhibit only H-2kb, whereas recipients (BDF) exhibit H-2kb/d.

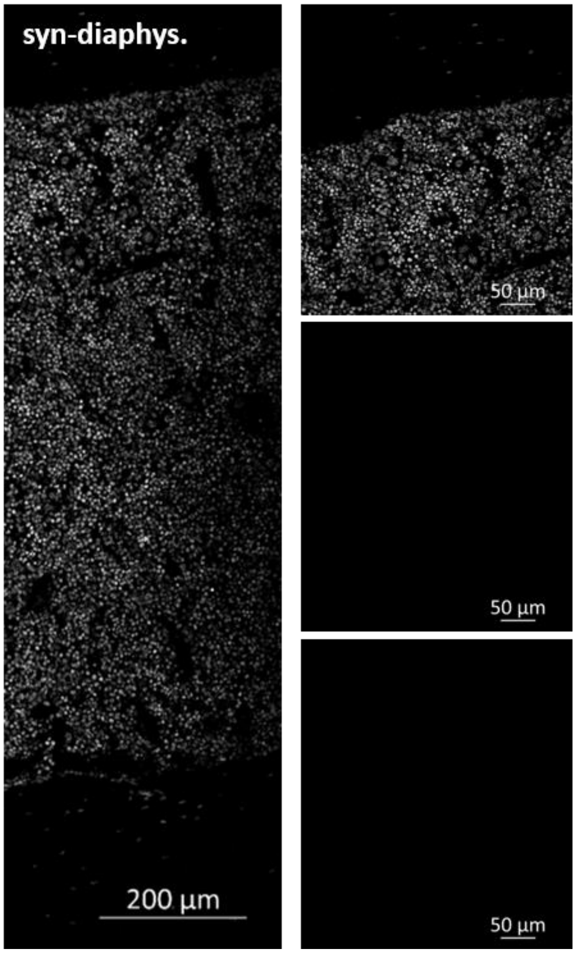

Supplementary Figure S2. Control images for the immunofluorescence staining. Femora harvested 20 days after syn-HSCT were chemically fixated, cryo-embedded, sectioned and stained with immunofluorescence antibodies. Sections were stained with DAPI (1st outtake image on the right side). To verify non-binding properties of the secondary antibodies we didn´t stained with anti B220 antibody (2nd outtake image on the right side) and for the anti-Osteocalcin staining, only the secondary antibody (Donkey anti-rabbit AlexaFluor 647) was used (3rd outtake image on the right side). Representative images are shown.

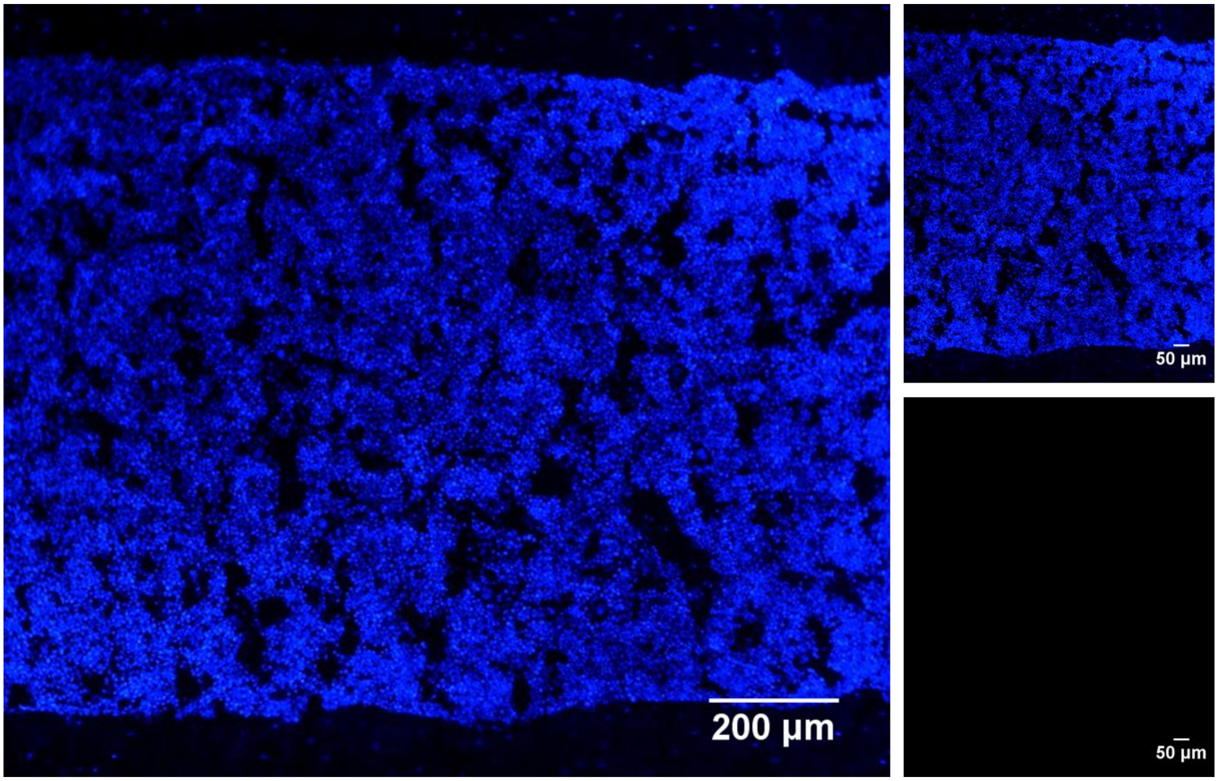

Supplementary Figure S3. Negative control for Endomucin staining. Bones were harvested on day +20 after syn-HSCT and cryo-sections were immunofluorescently stained. Control staining was performed with the secondary antibody only, without prior staining against Endomucin. The left picture shows a merged image of DAPI and the secondary antibody signal, the upper right picture shows the DAPI channel, the lower right image shows the endomucin channel (secondary antibody signal).
